# Nanostructuration of YAG:Ce Coatings by ZnO Nanowires: A Smart Way to Enhance Light Extraction Efficiency

**DOI:** 10.3390/nano12152568

**Published:** 2022-07-26

**Authors:** Nehed Amara, Aubry Martin, Audrey Potdevin, François Réveret, David Riassetto, Geneviève Chadeyron, Michel Langlet

**Affiliations:** 1 CNRS, Grenoble INP, LMGP, Institute of Engineering, Université Grenoble Alpes, 38000 Grenoble, France; amara.nehed@gmail.com (N.A.); aubry.martin@sigma-clermont.fr (A.M.); david.riassetto@grenoble-inp.fr (D.R.); 2 CNRS, Clermont Auvergne INP, ICCF, Université Clermont Auvergne, F-63000 Clermont-Ferrand, France; francois.reveret@uca.fr (F.R.); genevieve.chadeyron@sigma-clermont.fr (G.C.)

**Keywords:** light extraction, ZnO nanowires, phosphors, nanostructuring

## Abstract

In this study, we report on the enhancement of the light extraction efficiency of sol–gel-derived Y_3_Al_5_O_12_:Ce^3+^ (YAG:Ce) coatings using ZnO nanowire (NW) arrays. The ZnO NWs were grown by hydrothermal synthesis from a ZnO seed layer directly deposited on a YAG:Ce coating. Highly dense and vertically aligned ZnO NW arrays were evidenced on the top of the YAG:Ce coating by electron microscopy. A photoluminescence study showed that this original design leads to a different angular distribution of light together with an increase in emission efficiency of the YAG:Ce coating upon blue excitation, up to 60% more efficient compared to a non-structured YAG:Ce coating (without NWs). These improvements are ascribed to multi-scattering events for photons within the structure, allowing them to escape from the phosphor layer by taking optical paths different from those of the non-structured coating. This strategy of light extraction enhancement appears to be very promising, since it uses soft chemical processes and cheap ZnO NWs and is applicable to any sol–gel-derived luminescent coating.

## 1. Introduction

In recent years, the research on high-brightness, highly efficient and possibly highly directional Light-Emitting Diodes (LEDs) has been stimulated by the commercial application of lighting technology to display, illumination or even communication (Li-Fi) devices. In particular, currently commercialized white LEDs generally associate a semiconductor chip emitting in the ultraviolet or blue wavelength region to one or more phosphor(s) deposited in the form of coatings usually using either on-chip or remote configurations [1,2,3]. Even if present LED-based display and lighting devices are characterized by good photometric parameters, they still face technical challenges, among which are their luminous efficacy as well as their lack of light directionality due to the isotropy and incoherence of LED radiation [4,5,6]. These drawbacks generally require the use of bulky optics in LED devices to be corrected, which generates additional costs and technical intricacy. Furthermore, the devices still suffer from light loss due to photon backscattering related to the total internal reflection (TIR) phenomenon, entailing a limitation of Light Extraction Efficiency (LEE) [7,8,9], which represents the ratio between the number of photons escaping into the air and the number of photons generated in the Active Layer (AL) [5,7,10]. Together with Internal Quantum Efficiency (IQE), LEE is the second key parameter that determines the luminous efficacy of LED-based devices. IQE is mostly dependent on the quality of the AL (chip or phosphor), whereas LEE is mainly limited by the gap existing between the high refractive index of the chip (n~2.5 for blue emitting *n*-GaN) or the phosphor (n~1.84 for the most commonly used Y_3_Al_5_O_12_:Ce^3+^ phosphor [11]) and the air (n~1) [7,8]. The key to increasing the escape probability of photons, and in turn the LEE value, is to give them more chances to find the escape cone defined by θC=sin−1nairnAL, n_air_ and n_AL_ being the refractive indices of the air and of the AL (for example, the phosphor), respectively [12,13]. To reach this objective, several strategies have been developed and reported in the literature. They are based either on the angular randomization of photons by scattering them from a structured surface [12,13,14,15] or on using an intermediate layer with an appropriate refractive index between the AL and the air [4,16]. In both cases, the TIR will be attenuated and the LEE value will increase, which has been demonstrated on LED chips [5]. These mechanisms have been reported using surface roughening (roughened or corrugated substrates) [12,17,18], photonic crystals [19], nanorods or nanopillars [20,21], and many others. One can cite the study of Gorsky et al. [22] who recently studied YAG:Ce converters patterned with TiO_2_ nanodisks and measured a peak extraction enhancement by a factor 2 compared to a non-structured reference.

Among the cheap and eco-friendly potential materials to get LED improvement, ZnO has attracted much attention in recent years since 1. it can be easily obtained as tunable nanostructures, such as nanorods, nanopillars, nanowires and nanosheets [23,24]; 2. these nanostructures are characterized by an effective refractive index (n_effZnO_~1–1.8) intermediate between GaN-based LED (n~2.4) and air, expanding the escape cone value θ_C_ [25,26]; 3. the high surface-to-volume ratio of these nanostructures allows for better thermal dissipation, entailing a longer LED lifetime [27]; and 4. the surface nanostructuring induced by ZnO NWs or NRs gives rise to an increase in light scattering [28].

ZnO nanostructures have already been used to improve the emission efficiency or quality of GaN-based LEDs [26,27,28], but only a few reports [29,30,31] deal with their association with phosphors to yield an enhanced white light, whether in terms of light extraction or photometric parameters. Indeed, thanks to either their near-UV/blue emission or their large visible emission band, ZnO nanostructures can lead to a higher Color Rendering Index or more suitable color temperature [24,31]. In a very recent work [32], ZnO nanorods were decorated with perovskite quantum dots to produce a stable and color-tunable white emission, using a technical approach similar to the one we carried out in our previous study [31] to associate YAG:Ce nanoparticles with ZnO NW arrays. Additionally, among the several studies existing on the combination between YAG:Ce and ZnO, most of them imply ZnO micro or nanoparticles (not NWs or nanorods) blended with YAG:Ce to elaborate nanocomposites with either improved photocatalytic activity [33,34] or enhanced optical properties in terms of emission intensity or photometric parameters [35,36,37]. Hence, luminescent composite structures reported so far are different from the heterostructures we describe in this paper where, instead of being decorated by functional nanoparticles, ZnO NWs were directly grown from a seed layer deposited on a crystallized sol–gel-derived YAG:Ce(1 mol%) coating. Furthermore, ZnO NWs grown on phosphors through a chemical hydrothermal process have never been reported to our knowledge.

This strategy thus represents an innovation insofar as it allows for the easy structuring of any kind of phosphors available through the sol–gel process. The structural and morphological properties of ZnO alone, YAG:Ce alone and YAG:Ce/ZnO NWs heterostructures were studied by means of standard methods: X-ray Diffraction (XRD) and Scanning Electron Microscopy (SEM). Regular and angle-dependent photoluminescence measurements were carried out to determine the influence of the nanostructuring on the light extraction efficiency as well as on the emission angular distribution.

## 2. Materials and Methods

### 2.1. Elaboration of YAG:Ce Coatings

The first step in the elaboration of sol-gel derived YAG:Ce coating- consists in the synthesis of the YAG:Ce precur-sor sol. Such a precursor sol was prepared according to the method we already detailed for YAG:Tb matrix [38]. Anhydrous yttrium chloride YCl_3_ (99.99% pure, Aldrich, St. Louis, MO, USA), cerium chloride CeCl_3_ (99.99% pure, Aldrich), metallic potassium (98% pure, Aldrich), aluminium isopropoxide (99.99+% pure, Aldrich) and anhydrous isopropanol (iPrOH, 99.8+% pure, Aldrich) were used as starting materials. The synthesis consists of preparing separately two alcoholic solutions maintained under Ar flux: solution A made of anhydrous yttrium (2.97 eq.) and cerium (0.03 eq.) chlorides poured in anhydrous isopropanol and heated at around 85 °C to promote dissolution of cerium chloride beads; and solution B of potassium (9 eq.) isopropoxide resulting from the dissolution of potassium in isopropanol. After the overall dissolution of chlorides in solution A, solution B was slowly added to it under vigorous stirring: a precipitate of KCl immediately appeared. The mixed solution was maintained at 85 °C for 1 h. Then, a known quantity of aluminium isopropoxide (5 eq.) powder was poured directly into the A/B mixture. Since the final sol was intended to make coatings, it had to be chemically stabilized. Acetylacetone was used as a chelating agent for this purpose and was added to the previous mixture two hours after the aluminium precursor. The optimal quantity of acetylacetone to add (5 eq.) was previously determined in a specific study on YAG:Tb sols and powders [39]. After further reflux for 2 h at 85 °C, a clear and homogeneous yellowish solution (solution C) was obtained together with the KCl precipitate. The latter was removed by centrifugation after cooling. The obtained clear supernatant corresponds to the stabilized YAG:Ce precursor sol. In this work, the Ce doping rate of 1 mol% was chosen according to previously observed optimal luminescence performances under blue excitation (450 nm) [40].

Then, this stabilized sol was coated onto well-cleaned silica substrates using a dip-coating technique. After filtering with a 0.2 μm sieve, the solution was kept in a specifically designed Teflon® container. Afterwards, a 75 × 25 mm^2^ silica substrate was slowly dipped and withdrawn into the slightly viscous sol at a controlled speed of 8 cm/min. After coating, each layer was dried at 80 °C for 5 min in an oven to evaporate volatile organic compounds and then heat treated in a silica tube for 2 min at 400 °C to initiate the condensation process. Repeating this procedure, twenty multicoated amorphous films (precursors of YAG:Ce matrix) were achieved. A further sintering step at 1100 °C for 4 h was then used to proceed to YAG:Ce matrix crystallization leading to rather transparent crystallized coatings whose thickness was estimated to be around 400 nm based on previous studies [41]. These coatings were used as supports for the ZnO seed layer involved in the subsequent growth of ZnO nanowires. One of the samples was not combined with ZnO NWs and was used as a reference (YAG:Ce alone).

### 2.2. Growth of ZnO Nanowires

The hydrothermal growth of ZnO NWs requires the preliminary deposition of a crystalline ZnO seed layer. This was deposited using a sol–gel approach based on our previous work [42]. Briefly, a sol was prepared by diluting zinc acetate dihydrate (ZAD) and monoethanolamine (MEA) in 1-butanol with a ZAD concentration and a ZAD/MEA molar ratio fixed at 0.32 M and 1, respectively. After 3 h of stirring at 90 °C, a clear sol was obtained. Then, a 300 μL droplet of this sol was deposited by spin-coating at 3000 rpm on the previously prepared YAG:Ce coatings cut into pieces of 25 × 25 mm^2^. The obtained xerogel films were then annealed at 540 °C for 1h under air, leading to crystalline ZnO thin films. Finally, seed layers obtained in this way were cleaned with an oxygen plasma at a power of 12 W for 4 min to favor the subsequent growth of ZnO NWs.

For the second step, ZnO NWs were grown on the seed layers by hydrothermal synthesis at ambient pressure according to our previously published procedure using zinc nitrate hydrate (ZNH) and hexamine (HMTA) [43]. The 25 × 25 mm^2^ ZnO/YAG:Ce sample was fixed with a 45° tilt angle on a Teflon^®^ sample holder. Meanwhile, ZNH and HMTA were mixed at room temperature in 100 mL of deionized water with ZNH and HMTA concentrations of 25 mM. This mixture was stirred for 1 min before being heated up to 90 °C using a hot plate equipped with an automatic temperature regulation system. The sample holder (with substrate) was then immersed in the heated solution for 30 min, the coated side of the substrate being oriented downwards, in order to achieve growth of the NWs. Finally, samples were rinsed with deionized water and then dried with a nitrogen stream. A sample was elaborated using the same procedure but with a well-cleaned YAG:Ce-free silica substrate as a support layer for the ZnO seed layer and served as a reference for ZnO NWs alone.

### 2.3. Characterization Techniques

The X-ray diffraction (XRD) patterns of the coatings were obtained with a Philips Xpert Pro diffractometer operating with Cu-Kα1 radiation (λ = 1.5406 Å). XRD patterns were recorded on the angle range 10° < 2θ < 70° using a scan step (2θ) of 0.08°. The scanning electron microscope (SEM) images of the coatings were collected on ZEISS Supra 55 FEG-VP instruments at 2 MAtech. The observations were carried out under high vacuum at 3 kV and using a secondary electron detector (Everhart–Thornley detector). Prior to observation, the samples (coated silica slides) were attached to an adhesive carbon on one face.

Two different setups, represented via simplified schemes in Figures S1 and S2, were used to study the room-temperature optical properties of the samples. The first one (Figure S1) was a Jobin Yvon bench consisting of a Xe lamp operating at 400 W and two monochromators (Triax 550 and Triax 180) combined with a cryogenically cooled charge coupled device (CCD) camera (Jobin Yvon Symphony LN_2_ series). The resolution of this system is better than 0.1 nm. This setup was used to record emission spectra on the front side of the sample with an angle between the excitation source and the sample equal to 45°. The excitation wavelength was first set to 458 nm, since it corresponds to a blue commercial LED. The second optical mounting (Figure S2) was used to qualitatively and quantitatively characterize the influence of the nanostructuration created by ZnO NWs on guided light extraction. It allowed angle-resolved photoluminescence measurements by exciting the samples on the back side with a blue LED emitting at 458 nm. The luminescence signal was detected by an optical fibre mounted on a goniometer to collect emissions from 0° (front of the sample) to 90° with an angular resolution of 2°. The signal was then focused on the slit on a 32 cm focal monochromator and detected by a CCD camera. Measurements were made on both the YAG:Ce coating alone and the YAG:Ce/ZnO NWs heterostructure coating between 475 and 800 nm. Measurement conditions were optimized for each kind of sample and specific correction coefficients were used for the results to be quantitatively comparable.

## 3. Results and Discussion

### 3.1. Structural and Morphological Studies

**Figure 1:**
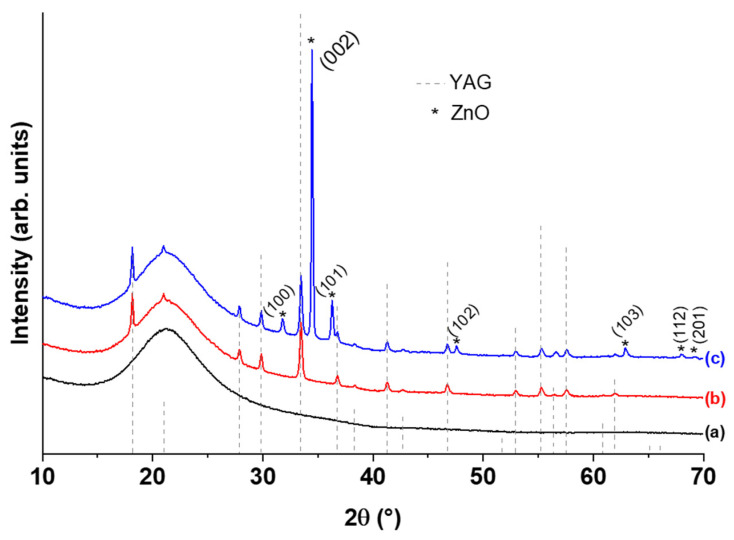
XRD patterns of (**a**) silica substrate, (**b**) YAG:Ce coating alone and (**c**) YAG:Ce/ZnO NWs heterostructure.

Figure 1 displays the XRD patterns of the YAG:Ce coating alone (Figure 1b) and of the YAG:Ce/ZnO NWs heterostructures (Figure 1c) compared with the pattern obtained for the silica substrate alone (Figure 1a). For all samples, a large signal related to the silica substrate around 21° (2θ) can be observed. The diffraction reflections of YAG:Ce alone (Figure 1b) are all ascribed to YAG matrix (JCPDS file 33-0040). These diffraction lines remain in the XRD pattern of the heterostructure (Figure 1c) associated with the main peaks of the hexagonal structure (wurtzite) of ZnO. In particular, the peak around 2θ = 34.8°, corresponding to the (002) plane of hexagonal ZnO, is by far the most intense, whereas the main diffraction peak of ZnO is generally (101) (JCPDS file 36-1451) [44]. This suggests that ZnO NWs have kept a mean c-axis orientation perpendicularly to the substrate as is generally obtained for hydrothermally synthesized ZnO nanorods or nanowires [45,46,47], even if they have grown on a YAG:Ce coating as a backing layer. However, one can note the fairly common presence of secondary reflections indicating a certain dispersion in this c-axis orientation. Furthermore, the diffraction peaks are very intense, which confirms the high crystallinity of ZnO NWs.

**Figure 2:**
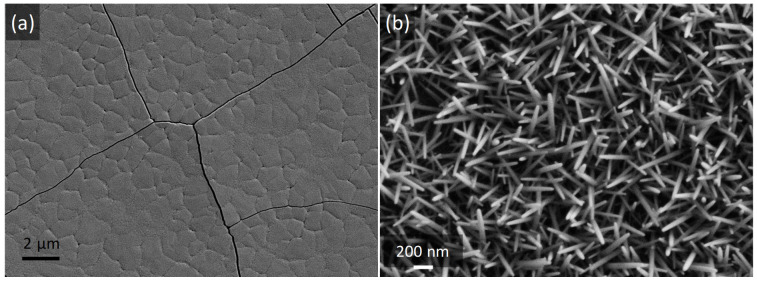
SEM images of (**a**) YAG:Ce coating alone and (**b**) ZnO nanowires grown from a ZnO seed layer deposited on a bare silica substrate.

SEM was used to observe the morphology of the heterostructure coatings and references. Top SEM views of YAG:Ce alone and ZnO NWs grown on the silica substrate are presented in Figure 2. The crystallized YAG:Ce coating (Figure 2a) exhibits a homogeneous and smooth “elephant skin” morphology with some cracks resulting from the coating sintering at 1100 °C. This morphology is the usual for crystallized sol–gel-derived YAG multi-coatings elaborated by dip-coating [41]. In Figure 2b, one can observe a part of the highly homogeneous array of ZnO nanowires that entirely cover the silica substrate. SEM confirms that, even if they are rather c-axis oriented (Figure 1b), the obtained NWs do not exhibit a perfect verticality. This behavior was already observed in our previous work [43]. NWs are characterized by diameters ranging between 30 and 50 nm and lengths from 400–500 nm (according to the cross-section SEM images shown in Figure S3).

**Figure 3:**
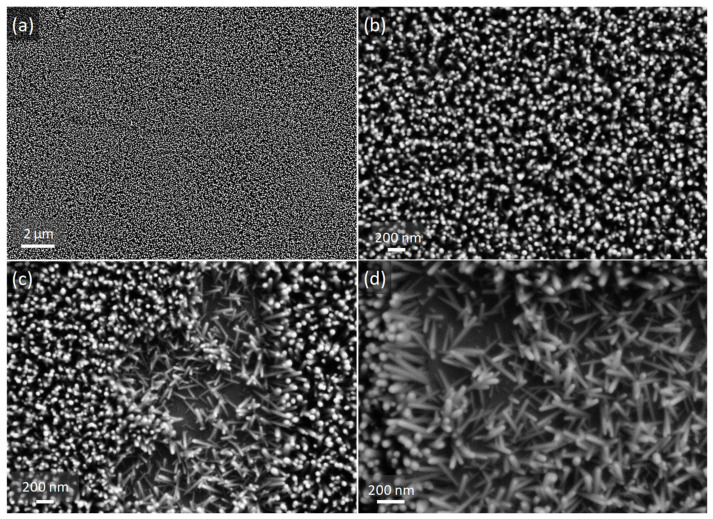
SEM images of YAG:Ce coating covered by ZnO NWs at different magnifications: (**a**) ×5000, (**b**,**c**) ×25,000 and (**d**): ×40,000. Image (**c**) and its zoom in (**d**) were recorded in one of the rare places where NWs had collapsed and hence are well distinguished.

Figure 3 gathers the top-view SEM images of the heterostructure coating at different magnifications. Figure 3a shows the high homogeneity of the ZnO NW arrays, despite the cracks of the YAG:Ce coating support. Indeed, the ZnO seed layer probably filled these cracks, leading to a smooth backing layer for the subsequent growth of ZnO NWs. Additionally, when comparing Figure 2b and Figure 3b, one can notice the higher density and enhanced verticality of ZnO NWs when they have grown on the YAG:Ce coating. Thanks to a local defect zone where NWs have collapsed (Figure 3c,d), they were measured and are characterized by diameters of around 30 nm and lengths of 300–400 nm. Hence, they are slightly thinner and shorter than those grown on silica substrate. These observations therefore demonstrate that the YAG:Ce coating noticeably influences the growth of NWs. The growth conditions of ZnO NWs or nanorods have been widely studied during the last two decades [48,49,50,51,52] and some parameters appear to be essential. Most of them are defined by the growth process (duration, pH, amount of capping agent, etc.). Here, all the growth conditions being equal otherwise, the only parameter responsible for these differences in ZnO NW arrays seems to be the seed layer morphology or its thickness.

Ding et al. [50], for example, showed in their study that the aspect of ZnO NW arrays was totally different depending on the substrate used to deposit the ZnO seed layer. This substrate determines the roughness and the thickness of the ZnO seed layer, which has a huge impact on the alignment of ZnO NWs and their density. Here, even if ZnO seed layers have not been specifically studied, we can guess that the seed layer deposited on the YAG:Ce coating is rougher than the layer covering the smooth silica substrate. Hence, it would offer more nucleation sites for ZnO NWs [49], leading to a higher density of more vertical, shorter and thinner NWs.

From SEM images and using ImageJ software, we assessed the area fraction of the coatings occupied by ZnO NWs. Since NWs tend to be vertical, this area fraction may be assimilated to the volume fraction. The analysis of several images led to an area fraction occupied by ZnO NWs that comprised between 40 and 50% of the coatings. This could have an important impact on the optical properties of the nanostructured coatings, as discussed in the next part. An example of the analyzed image is presented in Figure S4.

### 3.2. Optical Study

**Figure 4:**
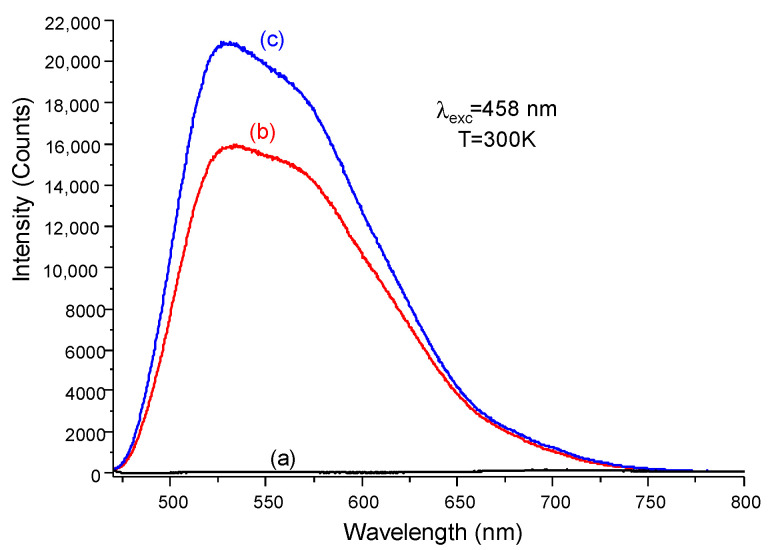
Room temperature emission spectra recorded under a 458 nm excitation for (**a**) ZnO NW arrays alone, (**b**) YAG:Ce coating alone and (**c**) YAG:Ce coating covered by ZnO NW arrays. Excitation source stroke in front of the sample at 45°.

Emission spectra of both YAG:Ce alone and heterostructure coatings, which were recorded at room temperature with an angle of 45° by exciting the samples via their front face (the face with the NWs for the heterostructure—see Figure S1), are presented in Figure 4b,c. They are compared with the emission spectrum of ZnO NWs alone (Figure 4a). The latter did not exhibit any luminescence when excited by a 458 nm radiation, which is a characteristic wavelength of commercial blue LEDs. This is in accordance with the excitation spectra presented in our previous paper [31]. The two other coatings are characterized by quite similar spectral profiles with broad asymmetric emission bands ranging from 475 to 750 nm. This profile is typical of Ce^3+^ ions in a YAG matrix—it is related to the electron transitions from the lowest crystal field splitting component of the 5d level to the ground state of Ce^3+^ (4f—^2^F_5/2_, ^2^F_7/2_) [53].

If the presence of ZnO NWs does not seem to influence the spectral distribution of YAG:Ce emission, it significantly enhances its intensity. Indeed, the area under the emission spectrum of the heterostructure was 1.3 higher than that of the YAG:Ce coating alone. This behavior was reproduced studying different areas of the samples and can result from two main reasons. The first would be an increase in the roughness of the YAG:Ce coatings when ZnO NWs are present, which offers many more possibilities for photons to find escape cones due to them being more numerous. This leads to a reduction in TIR phenomena occurring within the phosphor coating, and thus leads to an increase in light extraction, as already observed by Khan et al. [4] and Kim et al. [12]. The second would be the formation of a graded refractive index system thanks to the ZnO NW arrays. Indeed, the volume fraction of ZnO NWs was assessed as being inferior to 0.5 from the SEM images. Consequently, using the formula stemming from the effective medium theory [26,54] neff=[nZnO2×fZnO+nair21−fZnO]12, where n_ZnO_ and n_air_ are the refractive indices of bulk ZnO and air, respectively, and $f_{\mathrm{ZnO}}$ is the volume fraction of ZnO, we can roughly estimate the effective refractive index of this array to be $n_{\mathrm{eff}}\approx 1.59$ for yellow emission, which is concordant with the values found in the literature [25,26]. This is an intermediate value between that of bulk YAG:Ce (n ≈ 1.8) and that of air (n ≈ 1), leading to the aforementioned refractive index gradient. This gradient induces an enhanced LEE thanks to the increased value of the photon escape cone angle associated with a reduction in the Fresnel reflection at the interface between the heterostructure and air [26].

**Figure 5:**
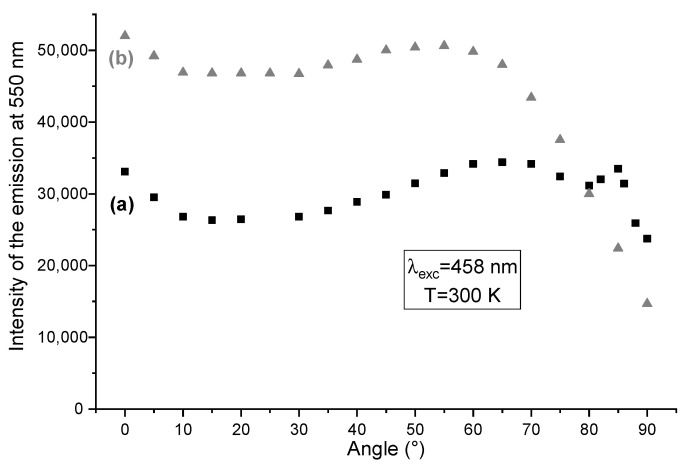
Room temperature angle-dependent emission intensity under a 458 nm excitation of (**a**) YAG:Ce alone and (**b**) YAG:Ce with ZnO NWs. Excitation source strikes the sample perpendicularly in its back (corresponds to 0°).

**Figure 6:**
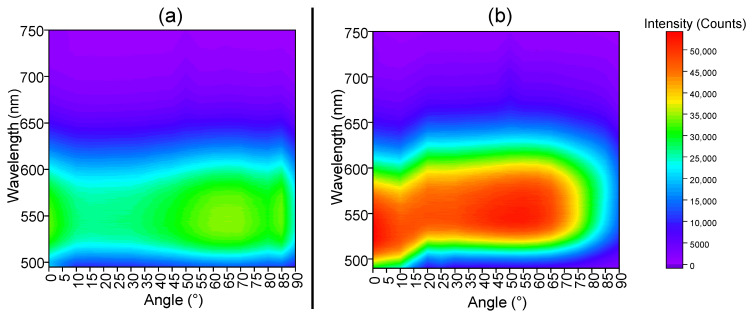
Luminescence maps corresponding to the emission spectra under a 458 nm excitation detected at different angle positions for (**a**) non-structured YAG:Ce and (**b**) nanostructured ZnO NWs/YAG:Ce coatings. The color code represents the emission intensity.

To study the effects of nanostructuring on the directionality of the photoluminescence, angle-resolved luminescence measurements were carried out on the same samples using the second setup described in the technical section (Figure S2). Emission spectral profiles tend to remain similar whatever the angle, as will be discussed below. To illustrate the impact of ZnO NW arrays on the emission efficiency, in Figure 5 we chose to represent the evolution of the emission intensity at 550 nm in relation to the measurement angle. In addition, Figure 6 represents the luminescence diagrams (color scale) corresponding to the emission spectra of the two samples as a function of the detection angle θ. The diagrams were recorded from θ = 0° to θ = +90° (0° corresponding to a light perpendicular to the layer) and between 490 nm and 750 nm. The higher the color temperature is, the stronger the emission intensity. Photons emitted below 490 nm are not recorded due to the use of a high-pass filter different from the filter employed in the previous setup.

From Figure 5 and Figure 6 one can observe, whatever the detection angle between 0 and 75°, the significant enhancement of the emission intensity when comparing the nanostructured YAG:Ce coating with the non-structured one, with both containing the same amount of phosphor. This is verified for a large range of emission wavelengths lying between 490 and 625 nm as shown in Figure 6. More precisely, between 0 and 60°, the YAG:Ce/ZnO NWs heterostructure is characterized by an emission 50 to 60% higher than that of YAG:Ce alone and the two curves present similar trends (Figure 5). This improvement may still be explained by both mechanisms detailed in Figure 4. For higher angles (superior to 65°), important differences can be noticed in the behavior of both samples according to Figure 5 and Figure 6. Indeed, the nanostructured YAG:Ce sample presents an important decrease in luminescence intensity, whereas the non-structured sample maintains a relatively high emission intensity until 90°.

**Figure 7:**
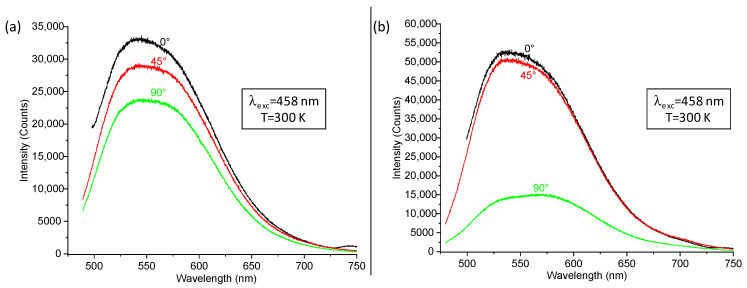
Room temperature angle-dependent emission spectra recorded for YAG:Ce (**a**) without and (**b**) with ZnO NWs under a 458 nm excitation. Excitation source strikes the sample perpendicularly in its back (corresponds to 0°).

These discrepancies are highlighted in Figure 7 where the emission spectra were plotted for both samples at three different angles: 0°, 45° and 90°. For measurements carried out at 0°, the signal corresponding to the excitation source still appears significantly for wavelengths between 475 and 500 nm, despite the use of the high-pass filter. This is the reason why spectra corresponding to 0° are plotted only from 500 nm. In the absence of ZnO NW arrays (Figure 7a), emission seems to progressively decrease from 0 to 90° and, at the highest angles, an important emission intensity was still recorded, which confirms a high number of guided modes within the phosphor layer. When ZnO NW arrays are present (Figure 7b), emission intensity remains stable between 0 and 45° but suffers from a considerable decrease at high angles. In particular, at 90°, the emission signal is much weaker, which could be related to the modification of the photon paths and to a drop in the number of guided modes. This is concordant with previous assumptions concerning the decrease in TIR phenomena (generally characterized by a high number of guided modes) with the improvement of light extraction.

This angle-dependent luminescence study shows that an increase in the number of extracted photons in the presence of ZnO NW arrays is associated with their spatial redistribution. Indeed, for the heterostructure, emission becomes preponderant between −70 and +70°, considering the light distribution symmetrical with respect to angle 0°. This feature is particularly interesting for foreseen applications, since controlling the light output angle is critical in LED-based devices.

## 4. Conclusions

In this work, we reported a simple way to elaborate luminescent YAG:Ce (1 mol%)/ZnO NW array heterostructures with improved emission properties compared with YAG:Ce (1 mol%) alone (with the same phosphor quantities). The design developed in this work significantly enhances light extraction thanks to the combination of two effects: a decrease in TIR, and the effective refractive index of the ZnO NW arrays, likely intermediate between YAG:Ce matrix and air, which allows for an increase in the angle of the escape cones. These mechanisms result in new optical paths for the photons to radiate out from the phosphor layer, and so on, in better light extraction than that of a non-structured YAG:Ce coating. This strategy is particularly interesting since: i. it involves the use of cheap and eco-friendly ZnO-based materials; and ii. it is assumed to be applicable to any kind of phosphor layer designed on a planar substrate for various optical devices.

## Data Availability

The data presented in this study are available on request from the corresponding author.

## Supplementary Materials

The following supporting information can be downloaded at: https://www.mdpi.com/article/10.3390/nano12152568/s1, Figure S1: Schematic representation of the configuration used to excite samples on their front side; Figure S2: Schematic representation of the setup used to carry out angle-dependent photoluminescence by exciting samples on their back side; Figure S3: Cross-sectional SEM view of the ZnO NWs grown from a ZnO seed layer deposited on a bare silicon substrate; Figure S4: Example of binarized SEM image used to assess the area fraction occupied by ZnO NWs within the NWs array using ImageJ software. NWs appear as the white part of the image.
