# Supplementary material for: Nanostructuration of YAG:Ce Coatings by ZnO Nanowires: A Smart Way to Enhance Light Extraction Efficiency
Source: Nanomaterials (Basel). 2022 Jul 26;12(15):2568. doi: 10.3390/nano12152568 (PMC9332156; doi:10.3390/nano12152568)
Supplement: Supplementary file 1 [file nanomaterials-12-02568-s001.zip › nanomaterials-1820297-supplementary.pdf]

Nanostructuration of YAG:Ce Coatings by ZnO Nanowires: A Smart Way to Enhance Light Extraction Efficiency

Nehed Amara [1,2], Aubry Martin [1,2], Audrey Potdevin [2,*], François Réveret [2], David Riassetto [1], Geneviève Chadeyron [2] and Michel Langlet [1,*]

[1] Institute of Engineering, Université Grenoble Alpes, CNRS, Grenoble INP, LMGP, 38000 Grenoble, France; amara.nehed@gmail.com (N.A.); aubry.martin@sigma-clermont.fr (A.M.); david.riassetto@grenoble-inp.fr (D.R.)
[2] Université Clermont Auvergne, CNRS, Clermont Auvergne INP, ICCF, F-63000 Clermont–Ferrand, France; francois.reveret@uca.fr (F.R.); genevieve.chadeyron@sigma-clermont.fr (G.C.)
* Correspondence: audrey.potdevin@sigma-clermont.fr (A.P.); michel.langlet@grenoble-inp.fr (M.L.)

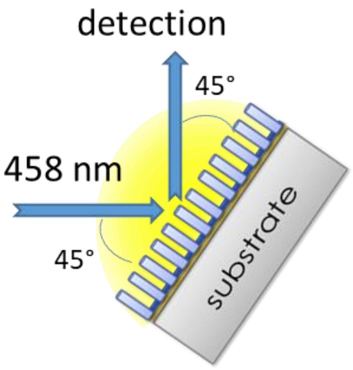

Figure S1. Schematic representation of the configuration used to excite samples on their front side.

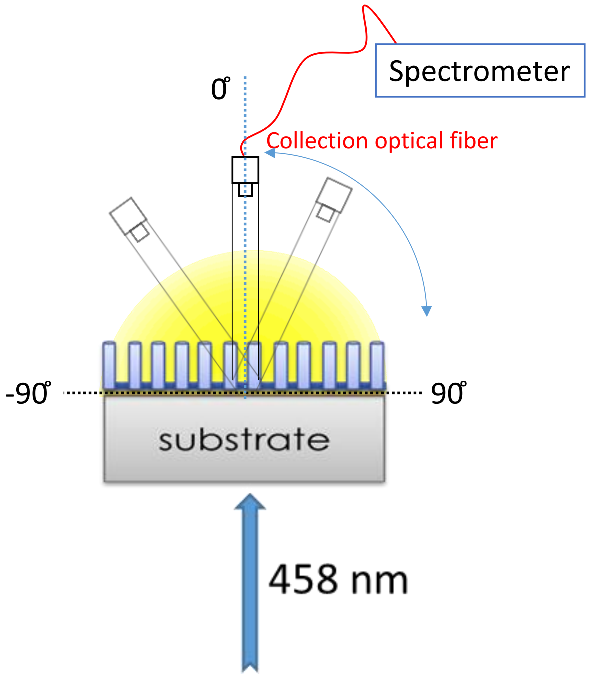

Figure S2. Schematic representation of the set-up used to carry out angle-dependant photoluminescence by exciting samples on their back side.

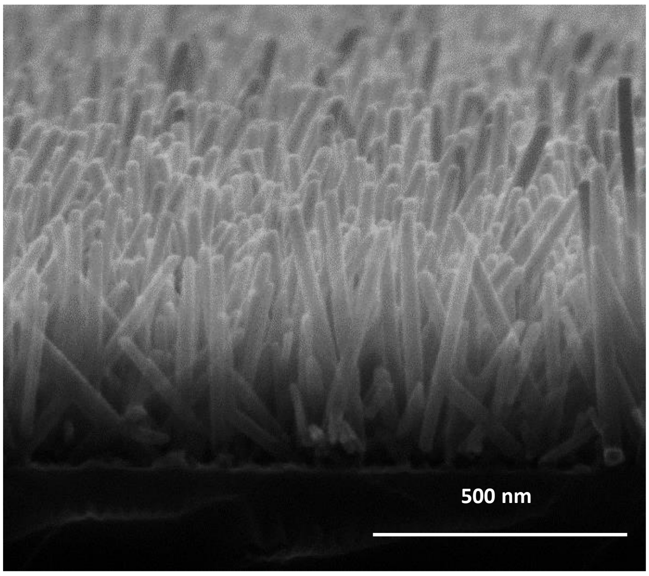

Figure S3. Cross-sectional SEM view of the ZnO NWs grown from a ZnO seed layer deposited on a bare silicon substrate.

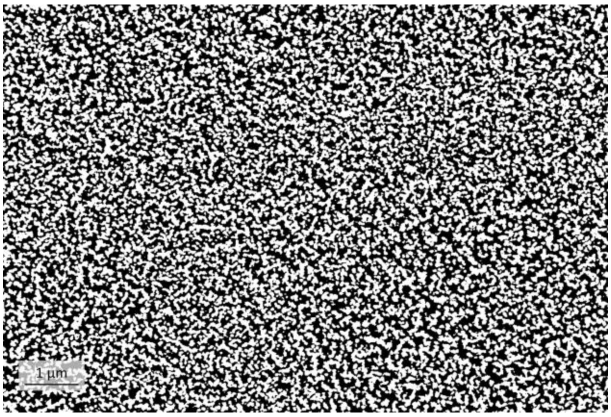

Figure S4. Example of binarized SEM image used to assess the area fraction occupied by ZnO NWs within the NWs array using ImageJ software. NWs appear as white part of the image.
